# Current Trends and Future Perspectives of Bradycardia, Renal Failure, Atrioventricular Nodal Blockade, Shock, and Hyperkalemia (BRASH) Syndrome: A Narrative Review

**DOI:** 10.7759/cureus.104731

**Published:** 2026-03-05

**Authors:** Toru Maruyama, Michinari Hieda, Mitsuhiro Fukata

**Affiliations:** 1 Cardiology, Haradoi Hospital, Fukuoka, JPN; 2 Cardiology and Nephrology, University of Occupational and Environmental Health Japan, Kitakyushu, JPN; 3 Hematology, Oncology, and Cardiovascular Medicine, Kyushu University Hospital, Fukuoka, JPN

**Keywords:** brash syndrome, covid-19, geriatric emergency, heart failure, pandemic

## Abstract

BRASH syndrome is defined as a clinical condition in which bradycardia, renal failure, atrioventricular (AV) nodal blockade, shock, and hyperkalemia interact to form a self-perpetuating negative spiral. Geriatric practitioners are increasingly likely to encounter elderly patients with this syndrome who are taking AV nodal blocking agents, such as calcium channel blockers (CCBs) or β-blockers. However, it remains unclear how the heart failure (HF) pandemic and coronavirus disease 2019 (COVID-19) have influenced the incidence, triggers, management, and clinical course of BRASH syndrome. Therefore, open-access databases were searched for publications from 1980 to 2025, identifying 41 eligible articles reporting a total of 54 patients with BRASH syndrome. The mean age of affected patients was 69.0 ± 15.1 years. Hypertension (HTN, 74%), chronic kidney disease (CKD, 61%), and diabetes (54%) were the most common comorbidities. More than half of the patients (52%) were prescribed angiotensin-suppressing agents (angiotensin-converting enzyme inhibitors (ACEi), angiotensin receptor blockers (ARB), or angiotensin receptor-neprilysin inhibitors (ARNI)) for HTN or HF. Two elderly patients were diagnosed with BRASH syndrome triggered by COVID-19. This literature review clarifies that BRASH syndrome commonly occurs in elderly patients with HTN or CKD and is often associated with everyday clinical events such as anorexia, vomiting, diarrhea, bleeding, and infection, including COVID-19. Our database search supports recognizing BRASH syndrome as an important clinical entity in geriatric emergency medicine. Geriatric practitioners should be aware of this condition to enable early diagnosis and appropriate management in the modern HF and post-COVID-19 era.

## Introduction and background

Providing effective, efficient, and reliable emergency care to aging populations is challenging. Geriatric visits to the emergency department (ED) continue to increase, and the field of geriatric emergency medicine is expanding rapidly, driven by the emergence of superaged societies in many countries. In the ED, geriatricians frequently encounter elderly patients with multimorbidity, in whom complex conditions interact synergistically and are further complicated by multiple medications (i.e., polypharmacy) [1-3].

An emerging but important topic in geriatric emergency medicine is BRASH syndrome, a newly recognized clinical entity characterized by bradycardia, renal failure, atrioventricular (AV) nodal blockade, shock, and hyperkalemia. These five components create a self-perpetuating negative cycle. The synergistic interaction between AV nodal blocking agents and hyperkalemia leads to bradycardia and shock, even in cases of mild hyperkalemia and therapeutic doses of AV nodal blockers. The resulting renal failure further impairs drug clearance and potentiates the toxicity of AV nodal blocking agents. This syndrome was conceptualized by Farkas et al. [4]. Elderly patients prescribed AV nodal blocking agents for hypertension (HTN) or atrial fibrillation (AF) may develop acute renal failure requiring hemodialysis or severe bradycardia requiring temporary pacing. In some cases, geriatric practitioners manage these patients in the ED without recognizing the presence of BRASH syndrome.

BRASH syndrome remains underdiagnosed and undertreated, partly because of its insidious onset and sudden clinical deterioration. Its incidence, triggers, optimal management, and clinical course remain unclear, particularly in the context of the modern heart failure (HF) pandemic and the post-coronavirus disease 2019 (COVID-19) era. These uncertainties prompted us to conduct a literature search for case reports and case series of BRASH syndrome and perform a descriptive analysis of the published data. Accordingly, we undertook a narrative review to better characterize BRASH syndrome in real-world settings and to provide clinically relevant information about this unique condition.

## Review

Methodology

Search Strategy

We searched multiple open-access academic indexing databases, including PubMed, Crossref, and Scopus, to identify reported cases of BRASH syndrome and to promote transparency and reproducibility of the literature search. Publications from January 1980 to December 2025 were included to cover the period of the COVID-19 outbreak. The final search was conducted at the end of January 2026. The search strategy used Medical Subject Headings (MeSH) terms that combined article types with all components of BRASH syndrome. The Boolean search string was as follows: “case report” OR “case series” AND “BRASH syndrome” OR “bradycardia” AND “renal insufficiency” AND “atrioventricular block” AND “shock” AND “hyperkalemia”. No search filters or limits were applied in order to minimize the risk of missing relevant articles and to broaden the scope of the review.

Selection of Literature

Regarding study eligibility, the inclusion criteria were English-language articles that provided a full description of the reported cases. The exclusion criteria included conference papers, press releases, poster presentations, meeting abstracts, and proceedings without a published full-text article. Our search strategy identified 41 eligible articles (32 case reports and 9 case series), comprising a total of 54 patients with BRASH syndrome after removal of duplicates [5-45]. The baseline characteristics of the included studies are presented in Table 1.

**Table 1:** Reported cases of BRASH syndrome. *Systolic but not mean blood pressure (mmHg). **Data after the initial treatment. ACEi: angiotensin-converting enzyme inhibitors, AF: atrial fibrillation, ARNI: angiotensin receptor/neprilysin inhibitor, BRASH: bradycardia, renal failure, atrioventricular (AV) nodal blockade, shock, and hyperkalemia, Ca: intravenous calcium administration, CABG: coronary artery bypass grafting, CKD: chronic kidney disease, COVID-19: coronavirus disease 2019, Cr: serum creatinine concentration, CRF: chronic renal failure, DL: dyslipidemia, DM: diabetes mellitus, HD: hemodialysis, HF: heart failure, HR: heart rate, HTN: hypertension, K: serum potassium concentration, mBP: mean blood pressure, NSAID: non-steroidal anti-inflammatory drugs, OMI: old myocardial infarction, nd: not described.

No.	Age	Sex	Clinical background	Drugs	Triggers	K (mEq/L)	Cr (mg/dL)	HR (bpm)	mBP (mmHg)	Treatment	Ref.
1	53	M	OMI	Verapamil, propranolol	Heat, exertion	6.8	1.6	32	70*	Isoproterenol, dopamine	[5]
2	50	F	CRF (renal transplant)	Verapamil, propranolol, quinidine	nd	7.4	ND	55	60*	Epinephrine, dopamine, Ca	[6]
3	75	F	HTN, CKD	Verapamil, captopril	nd	6.9	2.4	30	70*	Atropine, isoproterenol, Ca, pacemaker	
4	45	M	HTN	Propranolol, digoxin	Verapamil	7.8	ND	40	60*	Atropine, isoproterenol, dobutamine, Ca	
5	58	M	DM, CKD	Captopril, furosemide, triamterene	Verapamil	5.6	3.6	38	93	Fluid, atropine	[7]
6	66	F	HTN, CKD	Nitrendipine, bisoprolol	Verapamil, methyldopa	7.1	6.1	26	68	Fluid, atropine, isoproterenol, dopamine, Ca, sodium bicarbonate, insulin/glucose	
7	67	F	AF, CKD	Nifedipine, prazosin, digoxin, furosemide	Verapamil	nd	3.9	<20	nd	nd	
8	78	M	HTN, AF	Metoprolol, lisinopril	Anorexia	7.5	8.5	30	80	Fluid, furosemide, sodium bicarbonate, Ca, pacing	[8]
9	81	F	HTN, DM	Atenolol, digoxin, enalapril	nd	6	2.1	52	131	Fluid, furosemide, sodium bicarbonate	
10	57	M	nd	Carvedilol, digoxin, spironolactone, fosinopril	nd	6.8	2.7	48	73	nd	[9]
11	63	M	HTN, CRF (HD), liver transplant	Verapamil	Ciclosporin	6.8	HD	nd	nd	nd	[10]
12	57	M	CRF (HD)	Verapamil	nd	6.4	HD	nd	nd	Atropine	
13	58	M	HTN, CRF (HD)	Verapamil, ramipril, nicardipine	nd	6.7	HD	nd	nd	HD	
14	54	F	HTN, DM	Atenolol, diltiazem, irbesartan	nd	6.4	1.8	22	40	Fluid, Ca, insulin, pacing	[11]
15	70	M	HTN, DM, CKD	Metoprolol, enalapril, spironolactone	nd	6.5	3.3	44	71	Ca, albuterol, kayexalate, HD, pacing	[12]
16	78	F	HTN	ACEi, spironolactone, β-blocker, Ca antagonist	Potassium-rich fruits	7.9	2.1	33	nd	Fluid, Ca, insulin, kayexalate	[13]
17	77	M	CKD	Diltiazem, propranolol	nd	6.7	2.7	30	53	Fluid, dopamine, Ca, insulin/glucose	[14]
18	79	M	CKD	Metoprolol, amlodipine	Diarrhea, vomiting	6.4	4.4	28	79	Sodium bicarbonate, Ca, kayexalate, insulin/glucose	[15]
19	76	F	CKD	Carvedilol, spironolactone, ramipril	nd	9.2	1.3	28	79	Insulin/glucose, sodium bicarbonate, pacing	[16]
20	86	F	AF, HTN, CKD	nd	Dehydration	5.7	2.2	30	54	Fluid, atropine, pacing, insulin/dextrose	[17]
21	97	F	HTN, DM, CKD	Valsartan, amlodipine	nd	6.3	1.6	56	75	Fluid, Ca, insulin/glucose	
22	70	M	AF, HTN, DM, CKD	Valsartan, spironolactone, carvedilol	nd	6.1	2.1	38	62	Fluid, Ca, insulin/dextrose	
23	65	F	HTN, DM, CKD	Verapamil, valsartan	Urinary tract infection	5.6	3	48	85	Ca, insulin/dextrose	[18]
24	57	F	HTN, DM, CKD	Perindopril	Verapamil (add-on)	5.5	1.7	44	67	Ca, albuterol	
25	85	F	CKD	Sotalol, valsartan, spironolactone	Trimethoprim/sulfamethoxazole	10.1	2.5	33	61	Sodium bicarbonate, Ca, albuterol, HD insulin/dextrose	[19]
26	81	F	AF, HTN, DM, CKD	Amlodipine, bisoprolol, amiodarone	nd	5.8	2.8	33	104	Atropine, isoproterenol	[20]
27	24	M	HTN, HD	Metoprolol	Taking extra tablet of metoprolol	7.4	HD	40	nd	Fluid, atropine, Ca, sodium bicarbonate, epinephrine, pacemaker	[21]
28	51	M	Pituitary carcinoma, Cushing, HTN, DL, DM, hypothyroidism, CRF	Carvedilol, eplerenone	Trimethoprim/sulfamethoxazole	8.6	3.3	20	40	Insulin/dextrose, albuterol, pacing, Ca, hydrocortisone	[22]
29	88	F	AF, HTN, DM	Amlodipine, ranolazine	Nausea, vomiting	6.8	1.6	40	76	Fluid, Ca, albuterol, insulin/dextrose, atropine	[23]
30	62	F	HTN, DM, CKD	Carvedilol	Metolazone, bumetanide	8	4.1	31	42	Dopamine, Ca, insulin/dextrose, sodium bicarbonate	[24]
31	66	F	HTN, DM	Carvedilol, losartan	nd	6.2	2.2	35	71	Fluid, Ca, insulin/dextrose	[25]
32	43	F	AF, HTN, DM	Diltiazem, metoprolol	Anorexia	7.6	2.8	35	64	Fluid, Ca, norepinephrine, insulin/dextrose, sodium bicarbonate, pacing	[26]
33	55	F	HTN, DM, CKD	Diltiazem	nd	5.4	13.5	30-40	52	Ca, atropine, insulin/dextrose, albuterol, dopamine, kayexalate, pacing, HD	[27]
34	74	M	HTN, DM, CKD	Metoprolol, lisinopril	Diabetic ketoacidosis, anaphylaxis	7.1	3.1	40	40	Fluid, epinephrine, albuterol, Ca	[28]
35	81	F	HTN, DM, CKD	Atenolol, ramipril	NSAID	8.3	nd	29**	73**	Atropine, HD	[29]
36	nd	F	HTN, CKD	Carvedilol, verapamil	COVID-19	6.8	3	33	57	Atropine, dopamine, sodium bicarbonate, insulin/dextrose, Ca	[30]
37	69	M	HTN, DM	Metoprolol, lisinopril	nd	7.9	2.1	30	nd	Ca, epinephrine, insulin/dextrose	[31]
38	71	F	CKD	Octreotide	nd	6.5	nd	40-50	60*	Atropine, insulin, sodium bicarbonate, norepinephrine, vasopressin, epinephrine	[32]
39	62	F	HTN, DM	Atenolol, telmisartan, diltiazem	Vomiting, diarrhea	6.3	1.75	40	62	Dopamine, atropine, insulin/dextrose, adrenaline, Ca,	[33]
40	44	F	HTN, DM, CKD	Diltiazem, metoprolol, felodipine, spironolactone	nd	5.5**	2.57**	48	64	Atropine, dopamine, insulin/dextrose, Ca, pacing	
41	77	F	AF, HTN, DM,	Verapamil, lisinopril, furosemide	Urinary tract infection	5.6	3.4	30	56	Fluid, atropine, Ca, insulin/dextrose,	[34]
42	86	M	HF	Metoprolol, lisinopril	nd	6.7	2.8	30-40	nd	Fluid, Ca, insulin	
43	76	M	HTN, CKD	Amlodipine, atenolol	nd	7.3	3.7	26	53	Fluid, atropine, Ca, hydrocortisone, sodium bicarbonate, insulin/dextrose, epinephrine, pacing	[35]
44	58	M	AF	Lisinopril, carvedilol, sotalol	Urinary tract infection	6.5	2.2	30	50	Fluid, Ca, insulin/dextrose, dopamine	[36]
45	89	F	AF, HTN, DM	Amlodipine	Diarrhea	6.5	1.7	30	65	Fluid	[37]
46	83	F	HTN, DM, CKD	Bisoprolol, azilsartan	Ipragliflozin	7.8	3.3	24	52	Fluid, insulin, Ca	[38]
47	71	M	HTN, DM, CRF (HD)	Amlodipine, nifedipine, valsartan, carvedilol	Neck surgery	5.8	HD	31	54	Atropine, isoproterenol, Ca, insulin/dextrose	[39]
48	87	F	HTN, DM, DL	Verapamil, atorvastatin, gliclazide	Sepsis	5.6	1.8	42	61	Insulin/dextrose, Ca, albuterol	[40]
49	82	F	HTN, DM, CKD	Carvedilol, spironolactone, losartan	Anorexia	8.4	4.1	37	48	Ca, albuterol, insulin/dextrose, dopamine	[41]
50	67	M	AF, DM, CKD	Amlodipine, lisinopril, metoprolol, flecainide, hydrochlorothiazide	Golfing	5.6	2.1	24	91	Fluid, kayexalate insulin/dextrose	[42]
51	61	M	DM, CRF, HTN, DL	Carvedilol	COVID-19, skipping meal under insulin therapy	6.1	9.5	50	73	Fluid, atropine, dopamine, albuterol kayexalate, Ca	[43]
52	57	F	HTN, DM, CKD	Carvedilol, losartan, amlodipine	Missing HD	6	9.1	30	62	Fluid, atropine, dopamine, insulin albuterol, Ca, HD	[44]
53	94	M	HTN, CKD, HF, CABG	ARNI, nifedipine, eplerenone, bisoprolol	Gastric bleeding	5.8	1.8	44	64	Fluid	[45]
54	97	F	HTN, CKD	Amlodipine	Urinary tract infection	6.4	2	30	82	Fluid, pacemaker	

Statistics

All data were extracted from the full texts of the included articles. Extracted data were incorporated into descriptive analyses, and missing information was recorded as "not described (nd)." Descriptive statistics were used to calculate simple proportions (%) for categorical variables and to summarize continuous variables using the median, mode, or mean (± SD), with skewness reported where applicable. Analyses were performed using BellCurve in Excel, version 4.09 (Social Survey Research Information Co., Ltd., Tokyo, Japan). A team of three authors conducted the strategic meetings, literature search, data extraction, and descriptive analyses. 

Negative spiral in BRASH syndrome

Potassium and the Heart

BRASH syndrome is not simply a combination of bradycardia, renal failure, AV nodal blocking agent use, shock, and hyperkalemia; rather, these components interact to form a self-perpetuating negative spiral. Cardiac tissue, including the AV node, is highly sensitive to serum potassium (K^+^) concentration ([K^+^]), and electrical conduction through the AV node is mediated by voltage-dependent calcium channels. Hyperkalemia itself suppresses AV conduction because elevated [K^+^] makes the resting membrane potential of cardiomyocytes less negative, leading to steady-state inactivation of calcium channels. AV conduction block is therefore a key finding on the electrocardiogram (ECG) of patients with hyperkalemia [46].

Potassium Homeostasis

[K^+^] is tightly regulated through renal excretion in response to dietary intake and via shifts between intracellular and extracellular compartments. The renin-angiotensin-aldosterone system (RAS) promotes renal K^+^ excretion, and sympathetic stimulation increases renin release via β1-adrenoceptors, thereby activating RAS. Long-term β-blocker therapy can sustain [K^+^] by suppressing renin release and subsequent RAS activation [47]. Skeletal muscle, the largest organ in the body, serves as the primary storage site for intracellular K^+^. Vigorous muscle contraction releases K^+^ into the circulation and simultaneously activates the Na^+^-K^+^ ATPase on muscle cell membranes, facilitating K^+^ uptake back into cells. β-Adrenoceptors have long been recognized as key regulators of Na^+^-K^+^ ATPase activity [48,49]. Consequently, long-term β-blocker therapy can inhibit Na^+^-K^+^ ATPase activity, preventing post-exercise hypokalemia [50]. This mechanism may partly contribute to the onset of BRASH syndrome triggered by exercise or physical labor [5,21,42].

AV Blocking Agents

Calcium channel blockers (CCBs) and β-blockers suppress AV conduction, and their combined use can result in profound AV nodal suppression [51]. These agents also act synergistically with hyperkalemia to further inhibit AV conduction. In particular, verapamil can have deleterious hemodynamic effects when administered to patients with hyperkalemia and concomitant renal failure [6,40]. Hyperkalemia depolarizes the myocardium, which potentiates the inhibitory effect of verapamil on voltage-dependent calcium channels.

**Figure 1:**
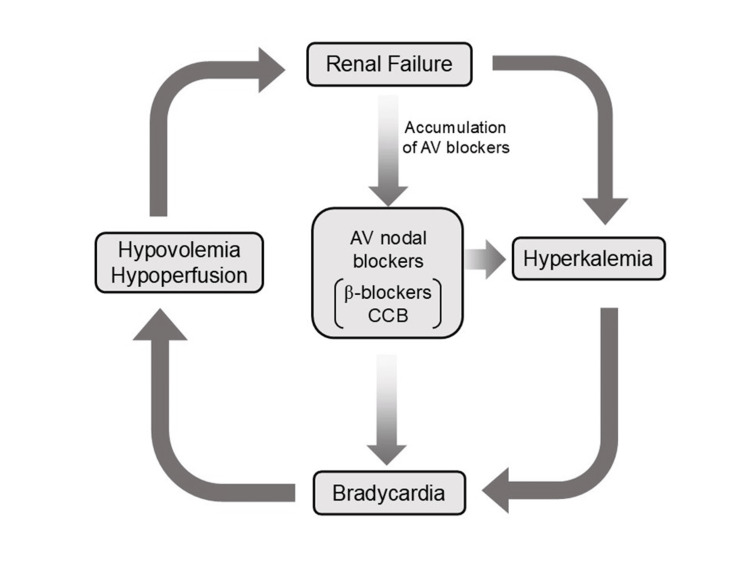
Schematic illustration of BRASH syndrome. BRASH: bradycardia, renal failure, atrioventricular (AV) nodal blockade (calcium channel blockers (CCBs) and β-blockers), shock, and hyperkalemia. The pathophysiology of BRASH syndrome is characterized by these components forming a self-perpetuating negative spiral. This schematic was adapted from reference [4] and modified for clarity (with permission) (created using Microsoft PowerPoint (Microsoft Corp., Redmond, WA, USA) and converted to JPEG).

The toxicity of AV blocking agents in the context of hyperkalemia leads to profound bradycardia and worsening hemodynamics, a process that is exacerbated by hypovolemia. Bradycardia and hypovolemia induce hypotension, acidosis, and systemic hypoperfusion, which further impair renal function, especially in patients with pre-existing chronic kidney disease (CKD). Impaired renal clearance also leads to the accumulation of AV blocking agents. Thus, hyperkalemia and accumulated AV blocking agents act synergistically, causing progressive bradycardia and creating a potentially lethal negative spiral (Figure 1).

Clinical profile of BRASH syndrome

Comorbidities and Triggers

**Figure 2:**
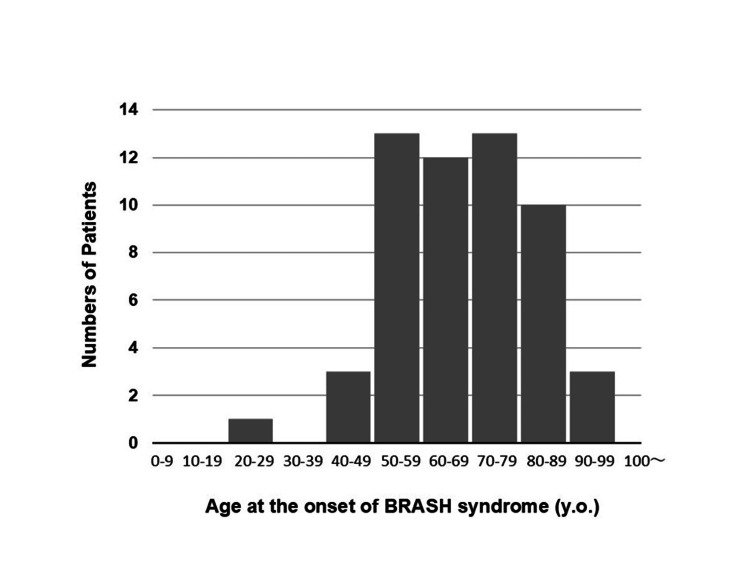
Age distribution at the onset of BRASH syndrome. The age of onset ranged from 24 to 97 years. The median and mean ages were 70 and 69.0 ± 15.1 years, respectively, with a negatively skewed distribution (skewness = -0.40). This figure was reconstructed from Table 1, excluding cases with missing data. BRASH: bradycardia, renal failure, atrioventricular nodal blockade, shock, and hyperkalemia.

Among the affected patients (N = 54), the median and mean ages at syndrome onset were 70 and 69.0 ± 15.1 years, respectively, with a range of 24-97 years. The age distribution showed a slight negative skewness (-0.40; Figure 2). There was a mild female predominance, with 31 females and 23 males. Younger patients with BRASH syndrome often had significant systemic diseases, such as cirrhosis [26], cancer [22], or renal insufficiency [21,33], and some required renal transplantation [6]. The most common comorbidities were HTN (74%), CKD (61%), and diabetes (54%). Triggers of BRASH syndrome included everyday events that cause dehydration or hypovolemia, such as diarrhea, vomiting, anorexia, infection, and bleeding. Additionally, incidental prescription or up-titration of nephrotoxic or potassium-elevating drugs in vulnerable patients also precipitated the syndrome [10,19,22,29]. These findings highlight that BRASH syndrome represents a distinct clinical entity in geriatric emergency medicine.

Bradyarrhythmias

**Figure 3:**
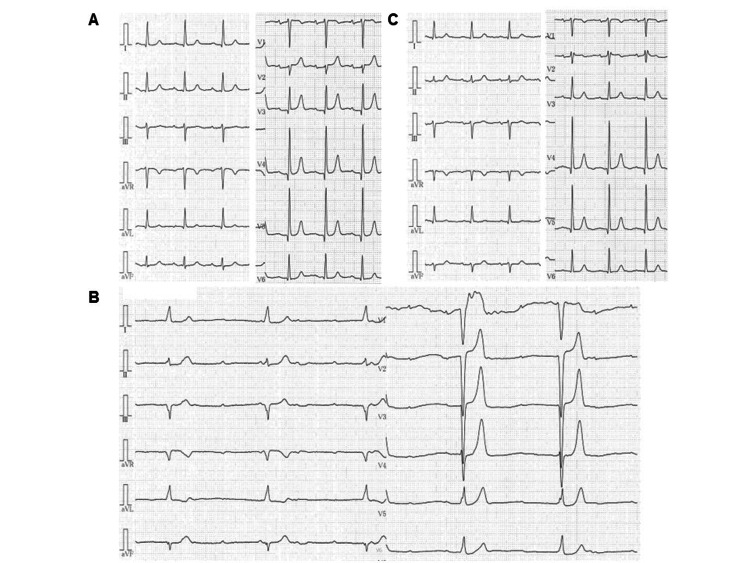
Serial electrocardiograms of a 97-year-old hypertensive female patient with BRASH syndrome. Panels (A), (B), and (C) show ECGs recorded before, immediately after, and one month after the onset of BRASH syndrome, respectively. Panel (B) demonstrates a complete AV block. Following permanent pacemaker implantation, the patient’s intrinsic P waves and QRS complexes are sensed by the device in panel (C), indicating that the complete AV block was reversible. These ECGs are from the first author’s patient [45], with informed consent obtained (unpublished data). BRASH: bradycardia, renal failure, atrioventricular nodal blockade, shock, and hyperkalemia.

**Figure 4:**
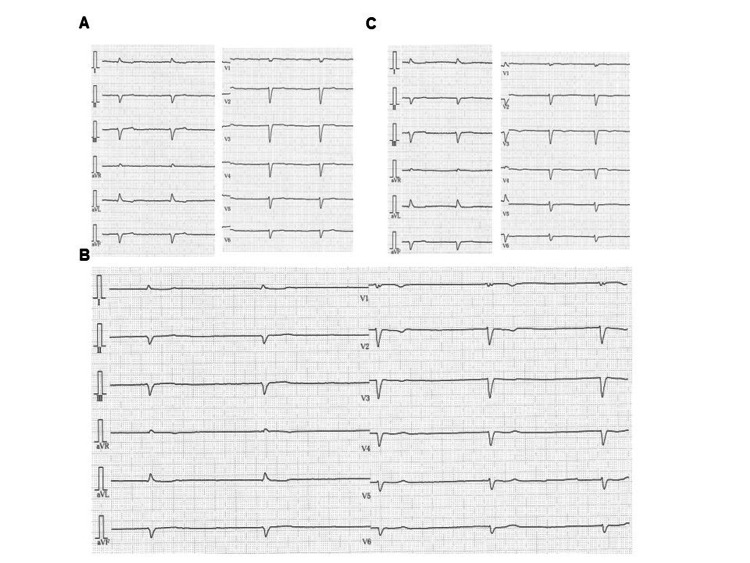
Serial electrocardiograms of an 89-year-old male patient with dilated cardiomyopathy presenting with BRASH syndrome. Panels (A), (B), and (C) show ECGs recorded before, immediately after, and one month after the onset of BRASH syndrome, respectively. The underlying rhythm is atrial fibrillation throughout, and the ventricular rate was reversibly reduced at the onset of BRASH syndrome. These ECGs are from the first author’s patient [45], with informed consent obtained from the patient’s relatives (unpublished data). BRASH: bradycardia, renal failure, atrioventricular nodal blockade, shock, and hyperkalemia.

Following the order of the BRASH acronym, we review each component narratively to illustrate the complete pathophysiology of this unique syndrome. Affected patients commonly presented with bradycardia, with a minimum heart rate (HR) at onset of 35.6 ± 8.8 bpm (range, 20-56 bpm). When recorded, ECGs showed profound sinus bradycardia (13%), sinus arrest (13%), junctional bradycardia (37%), or complete AV block (17%). Figure 3 and Figure 4 show representative ECGs from two cases, demonstrating marked but reversible bradyarrhythmias. Notably, ECGs in BRASH syndrome do not always display the typical features of hyperkalemia, such as peaked T waves, widened QRS complexes, or loss of P waves, likely due to preserved sino-ventricular (SV) conduction.

Renal Insufficiency and Causative Drugs

**Figure 5:**
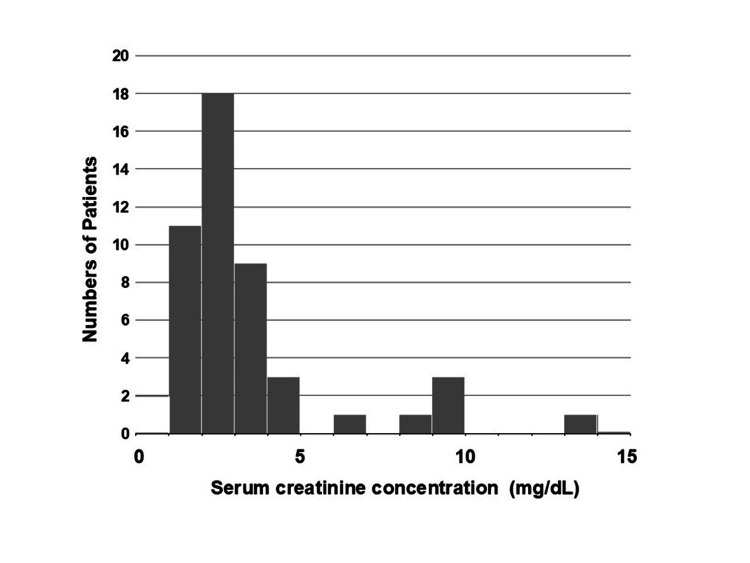
Distribution of serum creatinine at the onset of BRASH syndrome. Serum creatinine at onset ranged from 1.3 to 13.5 mg/dL (mean ± SD: 3.3 ± 2.4, n = 44), with a markedly positively skewed distribution (skewness = 2.61). Patients undergoing hemodialysis were excluded. This figure was reconstructed from Table 1, excluding cases with missing data. BRASH: bradycardia, renal failure, atrioventricular nodal blockade, shock, and hyperkalemia.

Bradycardia-induced hypoperfusion contributes to renal insufficiency. Although many patients had pre-existing CKD (61%) before the onset of BRASH syndrome, serum creatinine at presentation ranged from 1.3 to 13.5 mg/dL (mean ± SD: 3.3 ± 2.4 mg/dL, n = 44), with a markedly positively skewed distribution (skewness = 2.61) after excluding patients on hemodialysis (Figure 5). Regarding AV nodal blocking agents associated with BRASH syndrome, verapamil, a non-dihydropyridine CCB, was prescribed in 19% of cases, while dihydropyridine CCBs (nifedipine, nitrendipine, felodipine, and amlodipine) were used in 26% of cases. β-Blocker prescriptions reflected historical trends: propranolol was the classical agent reported in the earliest three case reports [5,6], whereas carvedilol (22%) and bisoprolol (7%) predominated in more recent cases. Combination therapy with a CCB and β-blocker, commonly used for HTN resistant to monotherapy, was observed in 33% of cases (Table 1) but can unpredictably cause profound hypotension and bradycardia even when each drug is at a therapeutic dose [47].

Hypotension and Hyperkalemia

**Figure 6:**
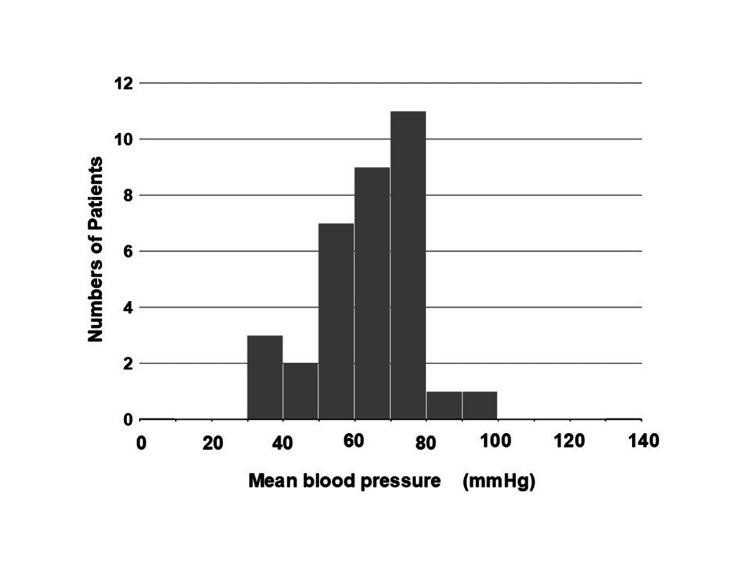
Distribution of mean blood pressure at the onset of BRASH syndrome. Mean blood pressure at onset ranged from 40 to 131 mmHg (mean ± SD: 66 ± 18 mmHg, n = 40), with a positively skewed distribution (skewness = 1.24), indicating that most, but not all, patients were hypotensive. Systolic blood pressure is not included. This figure was reconstructed from Table 1, excluding cases with missing data. BRASH: bradycardia, renal failure, atrioventricular nodal blockade, shock, and hyperkalemia.

**Figure 7:**
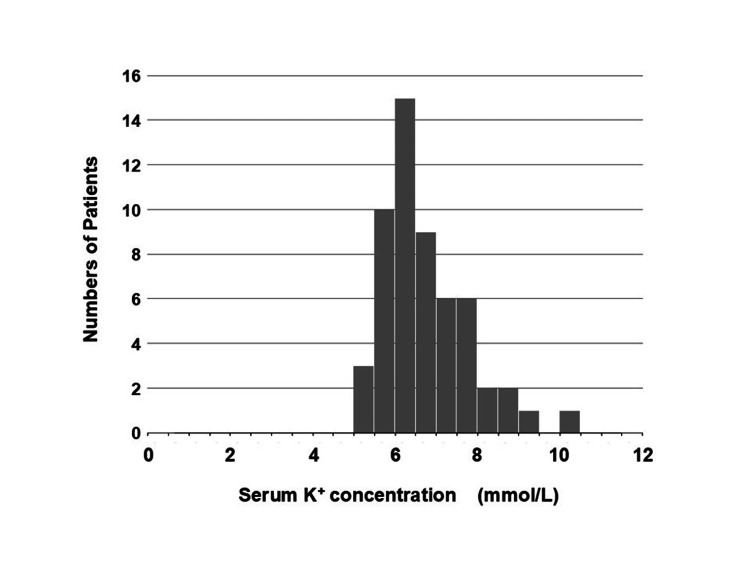
Distribution of serum potassium concentration at the onset of BRASH syndrome. Serum potassium (K^+^) concentration ([K^+^]) at onset ranged from 5.4 to 10.1 mmol/L (n = 52). The median [K^+^] was 6.6 mmol/L, the mode 6.8 mmol/L, and the mean 6.8 ± 1.0 mmol/L, with a positively skewed distribution (skewness = 1.03). Patients undergoing hemodialysis were excluded. This figure was reconstructed from Table 1, excluding cases with missing data. BRASH: bradycardia, renal failure, atrioventricular nodal blockade, shock, and hyperkalemia.

The hemodynamics of BRASH syndrome are characterized by hypovolemia, hypoperfusion, and marked bradycardia. In this review, patients’ systolic blood pressure at onset ranged from 40 to 131 mmHg, with a mean of 66 ± 18 mmHg (n = 40) and a positively skewed distribution (skewness = 1.24), indicating that most, but not all, patients were hypotensive (Figure 6). Normotensive patients may have had sufficient vasopressor compensation, which depends on factors such as patient characteristics, the trigger of BRASH syndrome, and the effects of causal drugs, particularly CCBs. Vasopressor responses may be preserved in younger patients but impaired in older patients with severe illness (e.g., sepsis or shock) or CCB intoxication. BRASH syndrome can occur even with mild hyperkalemia. In the reviewed literature, [K^+^] at onset ranged from 5.4 to 10.1 mmol/L (n = 52), with a median of 6.6 mmol/L, a mode of 6.8 mmol/L, and a mean of 6.8 ± 1.0 mmol/L (positively skewed, skewness = 1.03; Figure 7). These findings indicate that modest hyperkalemia, in the presence of AV nodal blocking agents, can trigger this unique syndrome, which likely explains why ECGs do not always show the classic features of hyperkalemia.

Treatment of BRASH syndrome

All patients with BRASH syndrome require urgent management. Regarding hemodynamics, acute fluid therapy is necessary to correct hypovolemia, and vasopressors should be administered to hypotensive patients. It is important to optimize the balance between intravascular volume resuscitation and vascular tone restoration, which varies depending on the underlying trigger of BRASH syndrome. For example, hypovolemia typically initiates the syndrome in cases of dehydration from vomiting, diarrhea, or bleeding, making fluid supplementation the first-line strategy. In contrast, vasodilatory hypotension associated with febrile infection or CCB intoxication requires early use of vasopressors.

In the literature, atropine was administered in 37% of cases but was largely ineffective, indicating atropine-resistant bradycardia. This is likely because the bradycardia in BRASH syndrome is not vagally mediated. Intravenous calcium stabilizes the myocardium, increasing HR and cardiac output, while intravenous epinephrine or inhaled albuterol can restore decompensated hemodynamics through positive inotropic and chronotropic effects and reduce hyperkalemia by activating Na^+^-K^+^ ATPase to shift potassium into muscle cells. Temporary or permanent pacing was required in 24% of cases, and emergent hemodialysis was initiated in 11% (Table 1). Although these interventions are effective for severe cases, early recognition and medical management remain essential to minimize the need for invasive procedures.

Current trends

HF Pandemic Era

HF is a leading cause of mortality, morbidity, and reduced quality of life in many industrialized countries. In the current era of the HF pandemic, several new pharmacological agents have emerged that reduce mortality. Accordingly, multiple guidelines recommend early initiation of the so-called “fantastic four” drugs for patients with HF with reduced ejection fraction: (1) β-blockers, (2) sodium-glucose cotransporter 2 inhibitors (SGLT2i), (3) mineralocorticoid receptor antagonists (MRA), and (4) angiotensin-suppressing agents, including angiotensin-converting enzyme inhibitors (ACEi), angiotensin receptor blockers (ARB), or angiotensin receptor-neprilysin inhibitors (ARNI) [52,53]. These agents have strong clinical evidence supporting their ability to reduce mortality, morbidity, and rehospitalization. However, caution is warranted when prescribing these drugs long-term in elderly patients with chronic HF and CKD. MRA, ACEi, ARB, and ARNI each carry a risk of hyperkalemia, and combination therapy, particularly MRA with ACEi, ARB, or ARNI, is often limited due to concerns about elevated potassium levels. In our review, many patients with BRASH syndrome had been prescribed angiotensin-suppressing agents (52%) or MRA (19%) (Table 1).

**Figure 8:**
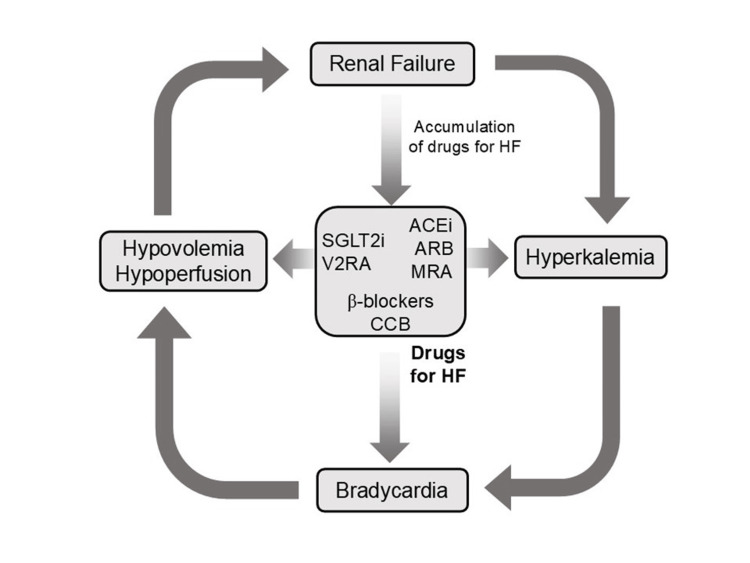
Schematic illustration of the pathophysiology of BRASH syndrome in the heart failure pandemic era. This original, hypothetical illustration depicts how contemporary heart failure (HF) therapies may contribute to BRASH syndrome. Angiotensin-converting enzyme inhibitors (ACEi), angiotensin receptor blockers (ARB), and mineralocorticoid receptor antagonists (MRA) can exacerbate hyperkalemia, while non-selective β-blockers and non-dihydropyridine calcium channel blockers (CCB) promote bradycardia. Tolvaptan (a vasopressin type 2 receptor antagonist, V2RA) and sodium-glucose cotransporter 2 inhibitors (SGLT2i) may induce hypovolemia, leading to renal failure and delayed drug clearance. Therefore, certain HF medications may contribute to each component of BRASH syndrome. This figure was created using Microsoft PowerPoint (Microsoft Corp., Redmond, WA, USA) and converted to JPEG format. BRASH: bradycardia, renal failure, atrioventricular nodal blockade, shock, and hyperkalemia.

New pharmacological options are emerging for patients with HF refractory to the “fantastic four” drugs. Ivabradine is a selective blocker of the hyperpolarization-activated cyclic nucleotide-gated (HCN) 4 channel, a pacemaker channel in the sinoatrial node. When added to guideline-recommended therapy, ivabradine can improve HF prognosis by reversing cardiac remodeling [54]; however, combined use with β-blockers increases the risk of bradycardia. Tolvaptan, a vasopressin type 2 receptor antagonist (V2RA), promotes free water clearance. SGLT2i offer substantial benefits for HF, CKD, and diabetes, but concurrent use of V2RA and SGLT2i may increase the risk of hypovolemia, particularly in elderly patients prone to dehydration. Vericiguat is a novel stimulator of soluble guanylate cyclase (sGC). In failing hearts, nitric oxide (NO) availability is reduced, sGC sensitivity to NO is decreased, and levels of sGC and cyclic GMP (cGMP) are diminished. Vericiguat is expected to restore NO-sGC-cGMP signaling and improve HF prognosis. AF often accompanies HF, and rate control is commonly attempted with CCBs and β-blockers. However, combining vericiguat with CCBs may cause excessive vasodilation and hypotension, leading to renal impairment and delayed drug clearance. While these agents do not directly induce BRASH syndrome, they can contribute to its development in vulnerable elderly patients with unstable hemodynamics worsened by hypovolemia from dehydration or bleeding (Figure 8).

Post-COVID-19 Pandemic Era

**Figure 9:**
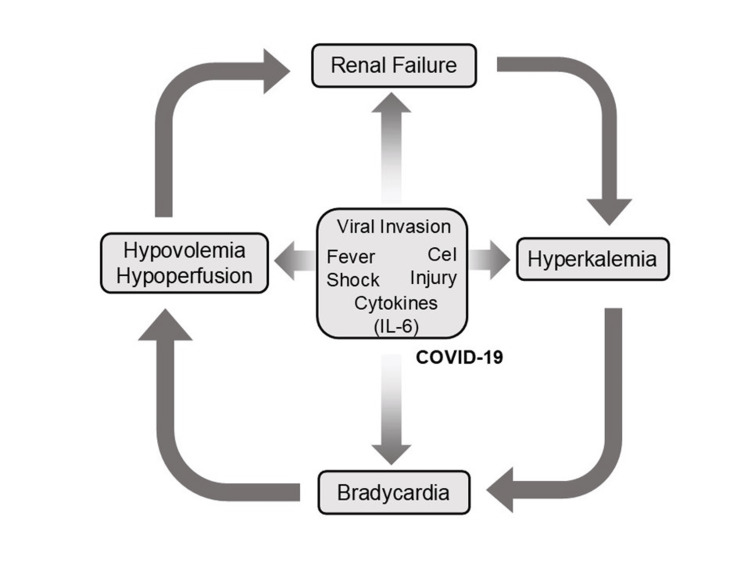
Schematic illustration of the pathophysiology of BRASH syndrome in the post-COVID-19 pandemic era. This original, hypothetical illustration depicts how infection with coronavirus disease 2019 (COVID-19) may contribute to BRASH syndrome. In COVID-19, fever and viremic capillary leakage can cause hypovolemia, hemodynamic deterioration, and shock. The infection affects multiple vital organs, including the kidneys, and inflammatory cytokines (interleukins, tumor necrosis factor-α, interferon-γ) contribute to acute kidney injury. Marked cytotoxic effects on somatic cells and renal impairment may result in hyperkalemia. Among these cytokines, IL-6 has been reported to suppress AV conduction [61], and the antiviral drug remdesivir may also induce bradycardia [62,63]. Therefore, both COVID-19 itself and its treatment may contribute to the negative spiral characteristic of BRASH syndrome. This figure was created using Microsoft PowerPoint (Microsoft Corp., Redmond, WA, USA) and converted to JPEG format. BRASH: bradycardia, renal failure, atrioventricular nodal blockade, shock, and hyperkalemia.

The COVID-19 pandemic has had profound effects on global science, culture, and the economy. Clinically, patients with COVID-19 exhibit a wide spectrum of acute symptoms, including fever, cough, and other upper respiratory manifestations. Severe cases may progress to acute respiratory failure, accompanied by profound hypoxemia and acidosis. In later stages, patients with severe COVID-19 often develop multi-organ failure, resulting in high mortality; acute renal failure is among the complications, although respiratory failure remains the primary concern [55-57]. In the post-COVID-19 era, new insights into BRASH syndrome have emerged. COVID-19 can trigger BRASH syndrome via multiple pathways. Fever and anorexia contribute to dehydration and hypovolemia, while direct viral effects, impaired hemodynamics, and elevated inflammatory cytokines (e.g., interleukins, tumor necrosis factor-α, interferon-γ) further impair renal function [58,59]. Resultant renal failure and massive somatic cell injury can induce hyperkalemia. In some patients, COVID-19 also suppresses cardiac AV conduction without evidence of acute coronary syndrome or fulminant myocarditis [60]. Inflammatory cytokines, particularly IL-6, may contribute to AV conduction block and bradycardia [61]. Additionally, remdesivir has been associated with bradycardia among antiviral therapies for COVID-19, as demonstrated in meta-analyses [62,63]. These processes (hypovolemia, hyperkalemia, and bradycardia) can interact to form the negative spiral characteristic of BRASH syndrome in COVID-19 patients (Figure 9).

To date, there have been two case reports of BRASH syndrome associated with COVID-19 infection. The first involved a 61-year-old male patient with end-stage renal failure whose BRASH syndrome was triggered by COVID-19 [43]. He had been on long-term carvedilol therapy, tested positive for COVID-19 during prehospitalization screening, and was successfully treated with emergent hemodialysis and temporary pacing before being discharged. The second case involved an elderly woman with CKD and hypertension who was taking aspirin, carvedilol, and verapamil. Upon presentation to the emergency department, she exhibited all components of BRASH syndrome and was confirmed COVID-19-positive after hospitalization [30]. These few reports may reflect underdiagnosis or underreporting of this critical condition, likely due to limited awareness. It is plausible that subclinical BRASH syndrome contributed to excess mortality during the COVID-19 pandemic. Regardless, early recognition and multidisciplinary management of elderly patients with BRASH syndrome remain essential, even in the post-COVID-19 era.

Limitations and future perspectives

**Figure 10:**
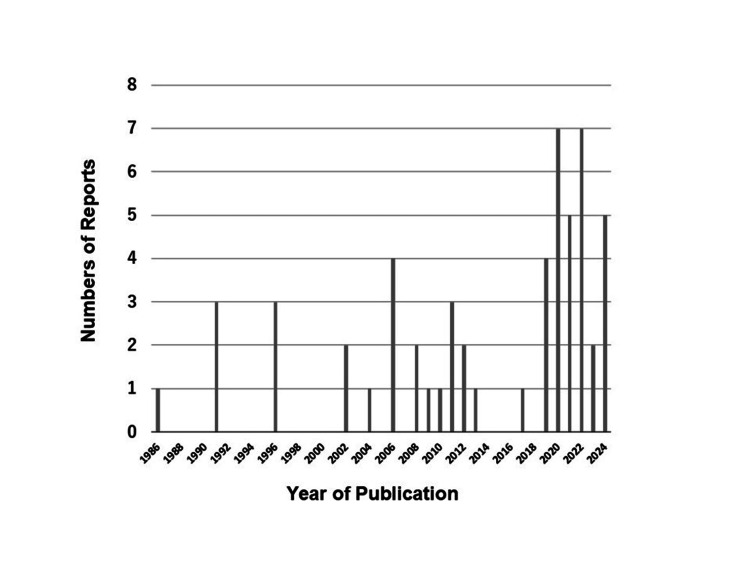
Distribution of publication years of case reports on BRASH syndrome. This figure shows the number of published case reports of BRASH syndrome by year, illustrating the publication trend over time. BRASH: bradycardia, renal failure, atrioventricular nodal blockade, shock, and hyperkalemia.

This literature review of BRASH syndrome has several limitations. First, this unique syndrome is likely underestimated due to under-recognition, underdiagnosis, or both. Elderly patients with BRASH syndrome may receive temporary pacing or hemodialysis in the ED without an accurate diagnosis. Consequently, underreporting in publicly available data is a key limitation. The publication trend shows only a modest increase over time, suggesting under-publication in the context of the HF and post-COVID-19 pandemic era (Figure 10). Part of this underdiagnosis stems from the absence of BRASH syndrome in the International Classification of Diseases 11th Revision (ICD-11) [64], as the syndrome must be distinguished from isolated hyperkalemia, renal failure, bradycardia, or cardiac drug toxicity [4]. Second, the case reports and series included in this review do not necessarily adhere to the case report (CARE) guidelines, which aim to improve accuracy, transparency, and utility [65]. Consequently, the articles are heterogeneous, and risk-of-bias or quality assessments were not performed. Looking ahead, pharmacologic triggers of BRASH syndrome are not limited to AV nodal blockers. Some cases in Table 1 involved drugs such as ranolazine [23], SGLT2i [38], or combinations like amiodarone [20] or flecainide [42] with β-blockers, recently described as “BRASH variants” [23]. Future research should integrate these variants into the broader framework of BRASH syndrome. To achieve this, a prospective, systematic review, rather than a narrative review, following the 2020 PRISMA guidelines [66], with protocol registration in PROSPERO [67], is warranted, as done in prior systematic reviews of this syndrome [34,68,69].

## Conclusions

BRASH syndrome is a newly recognized clinical entity in which bradycardia, renal failure, AV nodal blockade, shock, and hyperkalemia interact to form a negative spiral. Based on our database search, which identified 54 patients reported in 41 eligible open-access articles, BRASH syndrome appears to be more common in older, vulnerable patients, particularly those with HTN, CKD, or those receiving long-term treatment for HF with HR-lowering CCBs, β-blockers, MRA, and angiotensin-suppressing agents such as ACEi, ARB, and ARNI. Furthermore, COVID-19 may be linked to BRASH syndrome by inducing hypovolemia, shock, and renal failure, which can subsequently lead to hyperkalemia. However, the increase in publications on this syndrome has not been substantial, which may reflect possible underdiagnosis or underreporting in the current era of HF management and the post-COVID-19 pandemic.
